# Impact of Venetoclax Treatment Schedule on Hematologic Recovery and Treatment Response in AML Patients Unfit for Intensive Chemotherapy

**DOI:** 10.3390/cancers17071138

**Published:** 2025-03-28

**Authors:** Anja Schüpbach, Dilara Akhoundova, Ulrike Bacher, Henning Nilius, Michèle Hoffmann, Carlo R. Largiadèr, Yolanda Aebi, Michael Hayoz, Marie-Noëlle Kronig, Thomas Pabst

**Affiliations:** 1 Department of Medical Oncology, Inselspital, University of Bern, CH-3010 Bern, Switzerland; anja.schuepbach@students.unibe.ch (A.S.); dilara.akhoundovasanoyan@insel.ch (D.A.); michele.hoffmann@insel.ch (M.H.); marie-noelle.kronig@insel.ch (M.-N.K.); 2 Department of Hematology, Inselspital, University of Bern, CH-3010 Bern, Switzerland; veraulrike.bacher@insel.ch; 3 Department of Clinical Chemistry, Inselspital, University of Bern, CH-3010 Bern, Switzerland; henning.nilius@insel.ch (H.N.); carlo.largiader@insel.ch (C.R.L.); yolanda.aebi@insel.ch (Y.A.); michael.hayoz@insel.ch (M.H.); 4 Center of Laboratory Medicine (ZLM), Inselspital, University of Bern, CH-3010 Bern, Switzerland

**Keywords:** venetoclax, acute myeloid leukemia (AML), pharmacokinetics, treatment duration, hematologic toxicity, efficacy, complete response rate

## Abstract

(1) Background: The combination of venetoclax and hypomethylating agents (HMAs) is a standard first-line regimen for acute myeloid leukemia (AML) patients unfit for intensive chemotherapy. Since venetoclax-HMAs are usually administered until progression and delayed hematologic recovery is one of the limiting toxicities, cyclic administration including 7–14-day breaks is recommended. However, whether longer venetoclax schedules lead to higher response rates and how venetoclax pharmacokinetics correlate with toxicity and efficacy remains unclarified. In this single-center retrospective study, we analyzed how venetoclax plasma levels and treatment duration impact hematologic toxicity and treatment responses. (2) Methods: We analyzed the safety and efficacy of venetoclax-HMA combination regimens in a cohort of AML patients unfit for intensive chemotherapy treated at our institution between June 2020 and September 2023. The primary endpoint was the correlation between venetoclax plasma levels or administration schedule with hematologic recovery after the first cycle. Secondary endpoints included the following clinical outcomes: correlation with complete response (CR) status, progression-free survival, and overall survival. (3) Results: Within our cohort of 75 AML patients, we found no correlation between venetoclax plasma peak and trough levels, or venetoclax treatment duration (≤ or >14 days), and hematologic toxicity. Patients receiving shorter venetoclax schedules (≤14 days) had similar CR rates compared to patients treated with longer schedules. (4) Conclusions: Our results suggest that shorter (≤14 days) venetoclax schedules may have no negative impact on tumor responses in AML patients receiving venetoclax and HMA combinations. However, prospective validation studies would be required to confirm these findings.

## Simple Summary

Venetoclax, combined with hypomethylating agents (HMAs), is a standard first-line treatment for acute myeloid leukemia (AML) patients unfit for intensive chemotherapy. However, treatment schedules used across institutions are highly heterogeneous, and an optimal venetoclax administration schedule remains unclear. Moreover, whether longer venetoclax schedules lead to improved tumor responses is still unclarified. In this study, we investigated how venetoclax plasma levels and treatment duration (≤14 days vs. >14 days) correlate with hematologic recovery and responses in a cohort of 75 AML patients treated at our institution. Our study found no correlation between the venetoclax plasma peak and trough levels or venetoclax treatment duration (≤ or >14 days) and hematologic toxicity. Relevantly, patients receiving shorter venetoclax schedules (≤14 days) had similar complete response rates as compared to patients receiving longer schedules. In line with previous reports, our results suggest that shorter (≤14 days) venetoclax schedules do not negatively impact treatment efficacy. However, prospective validation studies would be required to confirm these findings.

## 1. Introduction

Acute myeloid leukemia (AML) is a hematopoietic stem cell malignancy characterized by an aggressive disease course and high lethality despite intensive treatment. For fit patients, first-line curative-intended treatment includes intensive polychemotherapy-based induction treatment, followed by consolidation with autologous or allogenic hematopoietic stem cell transplantation, depending on initial risk stratification and the initial response to induction chemotherapy [1]. However, AML frequently affects elderly patients, with a median age at diagnosis of 68 years [2]. Advanced patient age and/or coexisting comorbidities frequently preclude an intensive chemotherapy induction due to the high risk of complications and treatment-related mortality [3]. Patients unfit for intensive induction therapy are usually treated—through a non-intensive approach—with a combination of hypomethylating agents (HMAs) (azacytidine or decitabine) or low-dose cytarabine (LDAC) and the B-cell lymphoma 2 (BCL-2) inhibitor venetoclax [4,5]. Venetoclax is a selective inhibitor of the anti-apoptotic protein BCL-2, which is overexpressed in many lymphoid and myeloid malignancies, and it is crucial for the survival of AML tumor cells and involved in treatment resistance [6,7]. As compared to intensive polychemotherapy, the combination of venetoclax and HMA or LDAC is significantly less toxic, enables disease control, and delays AML progression. Patients receiving venetoclax in combination with an HMA have shown complete remission rates of 67% [8,9,10]. Relevantly, previous studies have shown that venetoclax monotherapy results in only modest anti-AML activity with a shorter duration of responses [11]. On the contrary, the synergistic combination with HMA or LDAC led to significantly higher response rates and more durable remissions [12]. Moreover, under adequate monitoring, venetoclax-based regimens can be administered in an outpatient setting, with a positive impact on patients’ quality of life. Still, myelosuppression is one of the limiting toxicities, and efforts have been made to optimize the venetoclax treatment schedule in order to minimize toxicity while maintaining efficacy. Since first-line venetoclax-based combination regimens are usually administered until progression, cyclic administration including 7–14-day breaks to enable hematologic recovery is usually recommended. Moreover, whether longer venetoclax schedules lead to higher response rates and how venetoclax pharmacokinetics correlate with toxicity and efficacy remains unclarified. Treatment schedules used across distinct institutions are highly heterogenous, and, lately, venetoclax schedules have been shortened in the clinical practice from initially 28 days to 14 and even to 7 days in 28-day cycles [13]. In this study, we analyzed how venetoclax pharmacokinetics and the administration schedule correlate with hematologic toxicity and response rates. We hypothesized that longer administration schedules negatively impact hematologic recovery, with no additional benefit on AML tumor responses.

## 2. Materials and Methods

### 2.1. Patients

This single-institution retrospective study included 75 AML patients treated with venetoclax-based combination regimens at the University Hospital of Bern, Switzerland, between 22 June 2020 and 1 September 2023. Patients who received at least one cycle of venetoclax and had at least one plasma drug level determined were included.

### 2.2. Study Endpoints and Data Collection

**Figure 1:**
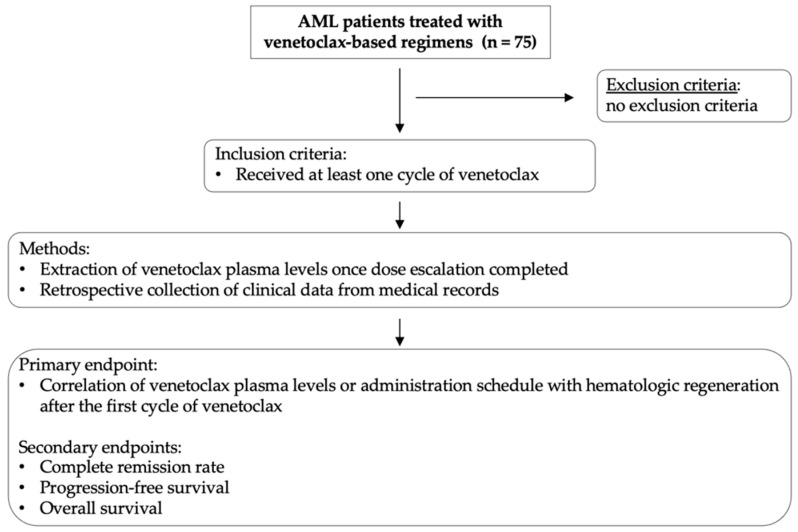
Schematic diagram summarizing study workflow and research methodology. Study inclusion criteria, brief summary of methods, and study endpoints are illustrated.

The primary endpoint of the study was the correlation between venetoclax plasma levels or administration schedule (≤14 vs. >14 days) with hematologic recovery after the first treatment cycle. The secondary endpoints included the following outcomes: complete remission (CR) rate, progression-free survival (PFS), and overall survival (OS). Risk stratification was performed according to the European Leukemia Network (ELN) 2022 guidelines [1]. For the purpose of this study, we included patients in CR with incomplete hematological regeneration within the CR group. Clinical data on patient baseline characteristics at first diagnosis, tumor responses, dates of progression and death, hematologic adverse events, as well as subsequent treatment lines were extracted from the clinical records. Study workflow and methods are summarized in Figure 1.

### 2.3. Venetoclax Plasma Levels

Blood samples for measurement of venetoclax peak and trough levels were collected on day 4 or later, once venetoclax dose escalation had been completed. Peak levels were collected approximately 6 to 10 h after venetoclax administration, and trough levels immediately before. Venetoclax plasma levels were measured using ultra-high performance liquid chromatography–tandem mass spectrometry (UHPLC) with multiple reaction monitoring (MRM). Briefly, following blood extraction, samples were stored at −80 °C. Before analysis, separate stock solutions of venetoclax ((venetoclax 95% solid form) Alsachim, Illkirch Graffenstaden, France) and the isotope-labeled analogue ([^2^H_7_]-venetoclax 98% in solid form from Alsachim, Illkirch Graffenstaden, France) were prepared: venetoclax and the internal standard [^2^H_7_]-venetoclax were dissolved at a concentration of 1 mg/mL in dimethyl sulfoxide/methanol (1:1, *v*/*v*). Calibrators were prepared based on a stock solution of venetoclax at 9.50 mg/L in methanol. In the same way, an independent stock solution was prepared for the quality controls. Seven calibrator spiking solutions were prepared by diluting the stock solution with methanol to final concentrations of 0.15, 0.30, 0.59, 1.19, 2.38, 4.75, and 9.50 mg/L (undiluted) for venetoclax. The same procedure was repeated for four quality control spiking solutions with the final concentrations of 0.22, 0.89, 2.97, and 7.42 mg/L in methanol. For protein precipitation and analyte extraction from calibrators and quality controls, 25 µL of the respective spiking solutions, at the appropriate concentrations, was added to 40 µL of a DC Mass Spect Gold serum (Golden West Biologicals, Temecula, CA, USA), followed by 180 µL of acetonitrile containing the internal standard [^2^H_7_]-venetoclax. For protein precipitation and analyte extraction from patient samples, 25 µL of methanol was added to 40 µL of the serum, followed by 180 µL of acetonitrile containing the internal standard [^2^H_7_]-venetoclax. After incubation and mixing for 10 min, the samples were centrifuged at 4000 RCF and 20 °C for 15 min. Then, 80 µL of the supernatant was diluted with methanol to a final volume of 240 µL. The prepared samples were sealed and stored in the autosampler at 10 °C until analysis.

For UHPLC-MS/MS analysis, 0.5 µL of the prepared samples were injected into a reverse-phase CORTECS UPLC T3 column of 120 Å, 1.6 µm, and 2.1 mm × 100 mm (Waters Corp., Milford, MA, USA), with a gradient mobile phase comprising 0.1% ammonium acetate with 1% formic acid (A) and acetonitrile containing 0.1% ammonium acetate with 1% formic acid (B). Each sample was resolved for 3.5 min at a flow rate of 0.5 mL/min with the linear gradient 0–1.2 min from 35 to 98% B; 1.2–2.0 min 98% B; and 35% B for 1.5 min. The column temperature was 30 °C. The eluent was introduced via electrospray ionization into the mass spectrometer (LC-MS 8060NX, Shimadzu Corp., Kyoto, Japan), operating in positive ion electrospray ionization mode (ESI+). The capillary voltage was set to 1.0 kV, and the Focus Voltage to 2.0 kV. The nebulizing gas flow was 2.5 L/min, the heating gas flow was 20 L/min, and the drying gas flow was 5 L/min. The interface temperature, desolvation line temperature, and heat block temperature were set to 400 °C, 150 °C, and 500 °C, respectively. MRM conditions were optimized by adjusting the collision energy to yield the most abundant product ions for each compound, which were subsequently used for MRM analysis (see summary of optimized MS/MS parameters given in Supplementary Table S1). Data analysis was performed with Labsolution Insight LCMS (version 3.8, Shimadzu Corp., Kyoto, Japan) by analyzing the areas under the specific MRM chromatograms compared to the area of the isotope-labeled analogue. The calibration curve was constructed using venetoclax concentrations ranging from 92.8 to 5937.5 µg/L and applying weighted linear regression with a weighting factor of 1/x.

### 2.4. Statistical Analysis

GraphPad Prism version 10 was used for statistical analyses and the graphical representations of the data. PFS was defined as the time from the first dose of venetoclax to the occurrence of progression, loss of follow-up, or death. OS was defined as the time from the start of venetoclax therapy to the date of death. Both PFS and OS curves were generated using the Kaplan–Meier method, with statistical significance assessed using the Mantel–Cox test and the Gehan–Breslow–Wilcoxon test. Categorical data were analyzed using Fisher’s exact test, and parametric data were evaluated using the unpaired *t*-test. Multiple regression analysis was conducted to explore statistical correlations between several independent variables and a single dependent variable. The following variables were included in the analyses: sex, age, European LeukemiaNet (ELN) risk stratification, French–American–British (FAB) classification, AML cytogenetics, selected molecular alterations, peripheral blood counts, and parameters related to venetoclax treatment (number and duration of cycles, dose, combination with HMA or LDAC, dose reductions, and selected co-medications), tumor responses, as well as time to progression and death. *p*-values were rounded to two decimals, and values below 0.05 were considered statistically significant. Percentage results were rounded to whole numbers.

## 3. Results

### 3.1. Patient Baseline Characteristics

The median age at AML diagnosis was 70 years, and men slightly outnumbered women at a ratio of 1.3. Fifty-six percent of patients had a primary AML, and forty-four percent had a secondary AML. Patient classification according to the FAB system is provided in Table 1. Based on the ELN risk classification, over 75% of patients were classified within the adverse risk group; moreover, nine (12%) patients were categorized as intermediate risk and eight (11%) patients as favorable. Thirty-one (41%) patients had a normal karyotype, fourteen (24%) had a complex or both a complex and monosomal karyotype, and twenty-nine (39%) patients had other cytogenetic abnormalities. In one (1%) patient, no data on karyotype were available. Most common mutations involved *ASXL1* (21%), *DNMT3A* (21%), *IDH2* (16%), *NMPM1* (20%), *RUNX1* (19%), *TET2* (24%), and *TP53* (17%). Patient characteristics at first diagnosis, including peripheral blood counts, are summarized in Table 1. The corresponding non-aggregated clinical data are listed in Supplementary Table S2.

### 3.2. Treatment with Venetoclax

Patients received a median of four cycles of venetoclax during the study period. Fourteen (19%) patients completed only one cycle, while 15 (20%) patients received more than 10 cycles. Fifty-one percent of patients received treatment cycles lasting 28 days, 31%, 42 days, and the remaining 19% received venetoclax with variable cycle durations. Almost three-quarters of the patients underwent venetoclax therapy in combination with azacitidine, while one-quarter was treated with decitabine or other combination drugs. Only 13% of patients received the standard venetoclax dose of 400 mg daily, while most patients (60%) received an adjusted dose of 100 mg due to the simultaneous intake of posaconazole or other CYP3A4 inhibitors. During treatment, dose reductions, with a median of 40%, were primarily applied to HMA or LDAC (35% of cases), whereas venetoclax dosing was mostly reduced by shortening the cycle duration to a median of 7 days (52% of cases) (Table 2). Treatment lines before and after venetoclax-based regimens are displayed in Supplementary Table S3.

### 3.3. Outcome of Venetoclax Therapy

**Figure 2:**
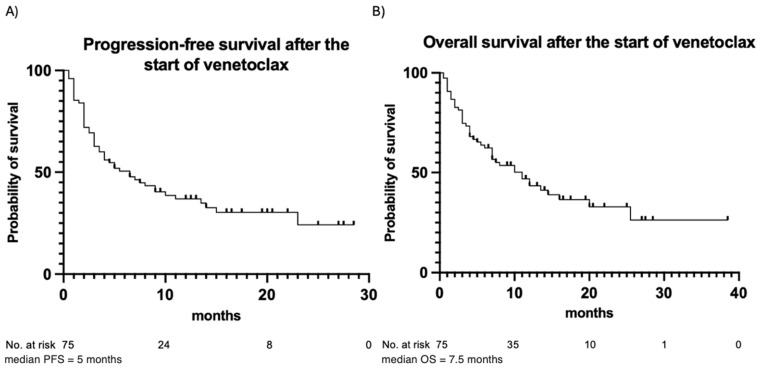
Progression-free survival (**A**) and overall survival (**B**) for the entire patient cohort. Observation time (months) is plotted in the X-axis, and the probability of survival, calculated using Kaplan–Meier analysis, is plotted in the Y-axis.

**Figure 3:**
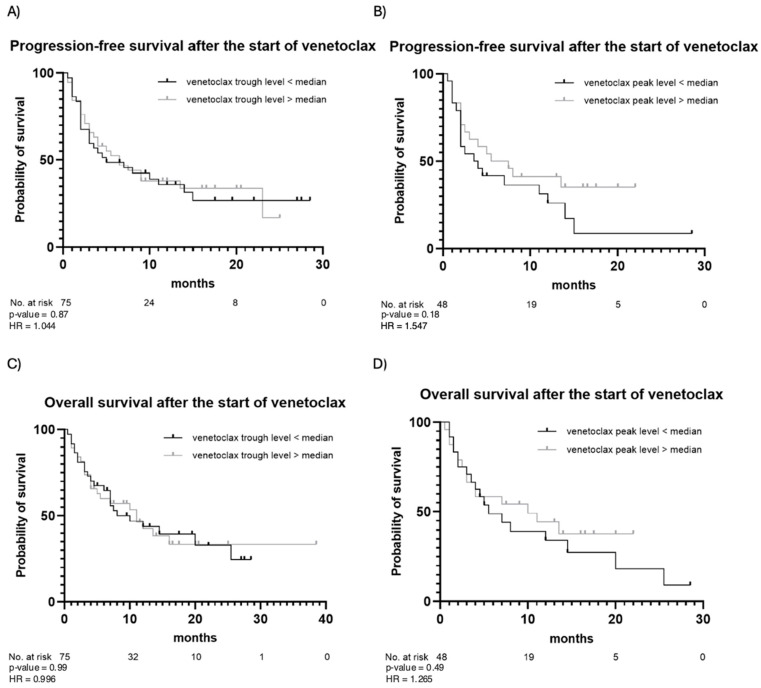
Progression-free survival and overall survival after the start of venetoclax stratified by venetoclax trough (**A**,**C**) and peak (**B**,**D**) levels. Observation time (months) is plotted in the X-axis, and the probability of survival, calculated using Kaplan–Meier analysis, is plotted in the Y-axis.

The outcomes of venetoclax therapy are summarized in Table 3. CR was achieved in 49 (65%) patients, while partial remission was observed in 10 (13%) patients. Fourteen (19%) patients had stable disease, and two (3%) patients died before response assessment. In the majority of patients (83%), the best response occurred following the first cycle of venetoclax. In the remaining cases, the best response was observed after the second (16%) or the third (1%) cycle. During the observation period, 50 (67%) patients experienced disease progression after a median of 5 months, and 45 (60%) patients died due to progression or disease-related complications. PFS and OS estimates are illustrated in Figure 2. In addition, PFS and OS curves for patients with trough and peak levels below vs. above the median are illustrated in Figure 3. No statistically significant differences in PFS or OS when stratifying by venetoclax plasma levels were observed. Patients with venetoclax trough levels below the median had a median PFS of 5 months, whereas patients with trough levels above the median showed a median PFS of 6.5 months (*p*-value = 0.87). The hazard ratio (HR) for this comparison was 1.044, with a 95% confidence interval (CI) from 0.599 to 1.818. The median OS was 10 months for patients with trough levels below the median and 11 months for those with trough levels above the median (*p* = 0.99), with an HR of 0.996 and a 95% CI from 0.555 to 1.787. A similar pattern was observed for both PFS and OS in patients with peak levels above and below the median. For PFS, the median was 3.75 months for patients with peak levels below the median and 6.5 months for those with peak levels above the median (*p* = 0.18). The median OS was 5.5 months for patients with peak levels below the median and 10 months for those with peak levels above the median (*p* = 0.49). Patients with a higher plasma peak concentration of venetoclax showed a trend toward slightly longer OS.

### 3.4. Drug Levels of Venetoclax

**Figure 4:**
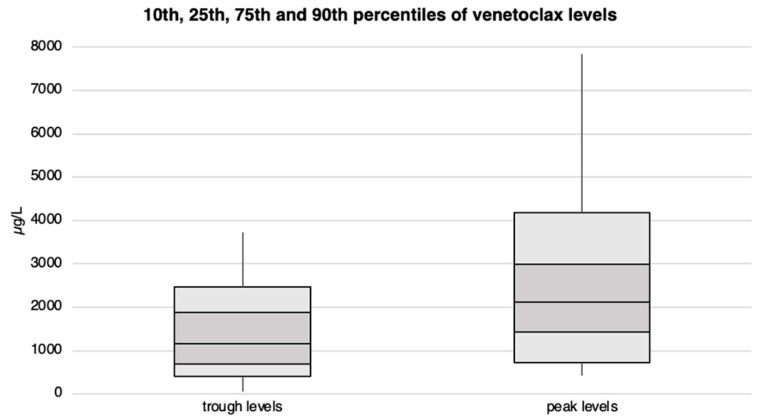
Venetoclax trough and peak plasma levels with corresponding percentiles for the entire patient cohort. The concentration levels (in µg/L) are plotted on the Y-axis, while the X-axis is divided into trough and peak levels.

Patients exhibited venetoclax plasma trough levels ranging from approximately 60 to 3’700 µg/L, with 50% of them reaching a minimum blood concentration of at least 1’170 µg/L. Peak levels varied from 400 µg/L to over 7’800 µg/L, with the median peak level slightly exceeding 2’100 µg/L. We observed no statistical differences in venetoclax plasma levels between patients receiving 100 vs. 400 mg/day. Venetoclax trough and peak levels are illustrated in Figure 4 and Supplementary Tables S4 and S7.

### 3.5. Impact of Remission Status and Venetoclax Plasma Levels on Hematologic Recovery

Data on the hematologic toxicities observed during the first and second cycle of venetoclax is illustrated in Supplementary Table S5. The correlation between remission status (CR versus not CR) or venetoclax plasma levels and hematologic recovery after the first cycle of venetoclax is illustrated in Table 4. Overall, 38 (51%) patients achieved CR after the first treatment cycle. Patients with CR more frequently had platelet levels over 100 G/L (*p*-value = 0.002) and neutrophil counts over 0.5 (*p*-value = 0.02), respectively, >1 G/L (*p*-value = 0.02) before starting the second treatment cycle, as compared to those who did not achieve a CR. Among all included patients, the median trough level was 1’220 µg/L, and the median peak level was 2’117 µg/L. No correlation was observed between the venetoclax plasma peak or trough levels and platelet levels above 100 G/L or neutrophil counts over 0.5/1 G/L before the initiation of the second treatment cycle.

Table 5 displays the correlation between neutrophil and platelet dynamics before and after the first treatment cycle CR status, as well as the venetoclax trough and peak levels. Twenty-four (33%) patients had platelet levels above 100 G/L, and 48 (67%) patients had levels below 100 G/L before starting venetoclax treatment. After the first cycle, platelets increased in 36 (50%) patients over 100 G/L, while in the other 36 (50%) patients, platelet counts either remained or had decreased below 100 G/L. Again, platelet recovery over 100 G/L was positively associated with the occurrence of CR (*p*-value = 0.02). Further correlations between venetoclax peak and trough levels, hematologic regeneration, and CR, as well as sex, dose of venetoclax, patient age, and the combined chemotherapy agent, are presented in Supplementary Tables S6–S10.

The correlation between venetoclax treatment duration and remission status (CR versus no CR) or hematologic recovery is shown in Table 6. Eighteen (25%) patients received venetoclax for 14 days or less, while the remaining 54 (75%) patients were treated for more than 14 days. CR rates were similar between the two groups, with no statistically significant difference observed (*p*-value > 0.99). No statistically significant correlation between shorter venetoclax treatment schedules (≤14 days) and the regeneration of platelet and neutrophil levels after the first cycle of venetoclax could be shown.

Table 7 displays the correlation between venetoclax treatment duration and change in neutrophil and platelet blood levels before and after the first cycle of venetoclax. No statistically significant difference between shorter (≤14 days) or longer (>14 days) venetoclax treatment schedules and change in platelet and neutrophil levels before and after the first cycle of venetoclax was found.

### 3.6. Factors Influencing Response, Hematologic Regeneration, and Venetoclax Trough and Peak Levels

CR, platelet, and neutrophil counts after the first cycle of venetoclax and venetoclax peak and trough levels were each considered dependent variables. Independent variables included age, sex, ELN risk stratification, de novo vs. secondary AML, dose of venetoclax, venetoclax trough and peak levels, response after the first cycle, and neutrophil and platelet counts before starting venetoclax treatment. Achieving a CR after the first cycle of venetoclax was significantly negatively associated with having an adverse ELN risk profile. Platelet counts below 100 G/L after the first cycle positively correlated with an adverse ELN risk profile, as did platelet counts below 100 G/L before starting venetoclax and trough levels exceeding the median. A negative correlation was observed between the lack of platelet regeneration and the achievement of a CR. Other regression analyses between the dependent and independent variables showed no statistically significant association (Table 8).

## 4. Discussion

The venetoclax and HMA combination is currently a standard first-line treatment for AML patients unfit for intensive chemotherapy. Given the favorable efficacy and safety profiles of these combinations, these regimens are being increasingly used in clinics. However, few data are available regarding the impact of venetoclax plasma levels on treatment response and hematologic toxicity. Moreover, while previous real-world reports have suggested that longer venetoclax schedules are associated with higher hematological toxicity, the consequences of venetoclax treatment duration on AML response rates remain unclarified. In this study, we aimed to assess the impact of venetoclax plasma levels and treatment duration on both hematologic toxicity and treatment efficacy. The pivotal phase 3 VIALE-A study assessed the efficacy and safety of the venetoclax–azacitidine combination in patients with a previously untreated AML who were unfit to receive intensive induction and consolidation therapy [8]. In this study, patients received venetoclax daily, following 28-day cycles. The results from this study highlighted a relatively high frequency of hematologic adverse events grade 3 or higher. Thrombocytopenia grade 3 or higher occurred in 45% of the patients, neutropenia in 42%, anemia in 26%, and febrile neutropenia in 42% [8]. Due to hematological toxicity, dose treatment interruptions, including the reduction in venetoclax treatment duration from 28 to 21 days, were necessary in 53% of patients [8]. Further studies showed that there is a need for venetoclax dose reduction in patients receiving concomitant treatment with strong CYP324 inhibitors, such as azole antifungals (e.g., posaconazol) [14]. Moreover, over the past years, individual reports on alternative venetoclax schedules have suggested that shorter administration schedules might overcome the handicap of delayed hematologic recovery without negatively impacting treatment efficacy [15]. Strikingly, the results of our study showed no correlation between the venetoclax plasma peak and trough levels and hematologic toxicity or treatment efficacy. Moreover, venetoclax treatment duration (≤ or >14 days) did not correlate with improved hematologic recovery. In line with previous reports, shorter venetoclax schedules did not lead to lower rates of CRs, suggesting a lack of negative impact on treatment efficacy [16,17]. The underlying biological mechanism remains unclarified [16,17]. Previous studies in AML and chronic lymphocytic leukemia showed a lack of impact of venetoclax exposure or peak levels on treatment responses [18,19]. However, the limitations of the current study include a single-center and retrospective design, a relatively small sample size, and a heterogeneous patient cohort. A relevant strength of our study is the consistency of observed CR rates with previous studies, including the phase 3 VIALE-A trial. In our study, CR was observed in 65% of patients, almost identical to the CR rate reported in the VIALE-A study (64.7%) [8]. Due to the common limitations of a retrospective study design, prospective validation studies would be required to more systematically assess the impact of the venetoclax schedule on AML tumor responses. In this sense, the ongoing observational multicentric VALOR study (NCT05215639) might provide more robust data on the safety and efficacy of venetoclax and HMA in a real-world patient cohort.

## 5. Conclusions

Our study could not show a significant correlation between venetoclax plasma levels or shorter vs. longer venetoclax administration schedules and hematologic toxicity in a cohort of AML patients unfit for intensive induction chemotherapy treated with venetoclax combined with HMA or LDAC. However, the results from our study suggest that shorter (≤14 days) venetoclax schedules may have no negative impact on AML tumor responses. Given the retrospective design of the current study, larger prospective studies would be required to confirm our findings.

## Figures and Tables

**Table 1:** Patient baseline characteristics.

	All Patients (n = 75)
Age at diagnosis, years (range)	70 (30–85)
Males/females (ratio)	43/32 (1.3)
ELN risk categories	
favorable, n (%)	8 (11)
intermediate, n (%)	9 (12)
adverse, n (%)	58 (77)
FAB classification	
M0, n (%)	8 (19)
M1, n (%)	11 (26)
M2, n (%)	11 (26)
M4, n (%)	9 (21)
M5, n (%)	3 (7)
Primary AML	42 (56)
Secondary AML	33 (44)
Cytogenetics	
normal karyotype, n (%)	31 (41)
complex karyotype, n (%)	4 (5)
complex and monosomal karyotype, n (%)	10 (13)
del(5q) or del(7q), n (%)	5 (7)
trisomy 8, n (%)	4 (5)
unknown, n (%)	1 (1)
others ^1^, n (%)	20 (27)
Mutations ^2^	
*ASXL1*mut, n (%)	16 (21)
*BCOR*mut, n (%)	6 (8)
*DNMT3A*mut, n (%)	16 (21)
*FLT3*mut, n (%)	10 (13)
*GATA2*mut, n (%)	6 (8)
*IDH1*mut, n (%)	6 (8)
*IDH2*mut, n (%)	12 (16)
*NPM1*mut, n (%)	15 (20)
*NRAS*mut, n (%)	10 (13)
*RUNX1*mut, n (%)	14 (19)
*SF3B1*mut, n (%)	9 (12)
*SRSF2*mut, n (%)	15 (20)
*STAG2*mut, n (%)	6 (8)
*TET2*mut, n (%)	18 (24)
*TP53*mut, n (%)	13 (17)
others ^3^, n (%)	41 (55)
no mutations, n (%)	3 (4)
Peripheral blood parameters	
leukocytes ×10^3^/µL, median (range)	4.9 (0.2–174)
platelets ×10^3^/µL, median (range)	65 (4–341)
hemoglobin g/L, median (range)	90 (37–132)
peripheral blasts %, median (range)	18 (0–92)
BM blasts %, median (range)	60 (20–95)
LDH U/L, median (range)	405 (156–6553)

^1^ One patient each with: t(12;14)(q24.3q11.2); t(1;3)(p36.3;q12); inv(3)(q21q26), −7; der(2)t(1;2)(q25–31;q31), der(7)t(7;8)(q31;q21); tetrasomy 8; i(14)(q10); dic(7;12)(p11.2;p11.2), add(17)(p13); inv(9)(p11q13); +1, der(1;7)(q10;p10); der(10), −7; del(7)(q22q3?4), del(20)(q11.2q13.2); t(10;11)(p12;q14); del(5)(q14q32), t(3;11)(p13;p15); t(8;21)(q21.3;q22.1); t(3;3)(q21.3;q26.2), del(7)(q22q36); inv(16)(p13.1q22); del(3)(q12q26), t(11;15)(q23;q12–14); t(6;11)(q27;q23.3); +13, +13; −7; ^2^ A patient can have more than one mutation; ^3^ ≥1 of the following mutations: *ASXL2*, *CBL*, *CEBPA*, *CTCF*, *CTNNA1*, *CUX1*, *DDX41*, *ETV6*, *EZH2*, *IKZF1*, *JAK2*, *JAK3*, *KRAS*, *NF1*, *NOTCH1*, *PHF6*, *PTPN11*, *RB1*, *SETBP1*, *SH2B3*, *SMC1*A, *SUZ12*, *U2AF1*, *WT1*, *ZBTB7A*, *ZRSR2*.

**Table 2:** Details on treatment with venetoclax.

Treatment with Venetoclax	All Patients (n = 75)
Number of cycles, median	4
1, n (%)	14 (19)
2, n (%)	12 (16)
3–4, n (%)	12 (16)
5–10, n (%)	22 (29)
>10, n (%)	15 (20)
Duration of cycles, days	
28, n (%)	38 (51)
42, n (%)	23 (31)
other cycle duration ^1^, n (%)	14 (19)
Combination of venetoclax with	
azacitidine, n (%)	55 (73)
decitabine, n (%)	5 (7)
other combination ^2^, n (%)	15 (20)
Dose (mg per day)	
100, n (%)	45 (60)
400, n (%)	10 (13)
other ^3^, n (%)	20 (27)
Relevant co-medication (CYP3A4 inhibitors), n (%)	63 (84)
posaconazol, n (%)	54 (86)
isavuconazol, n (%)	5 (8)
voriconazol or fluconazol, n (%)	4 (6)
Dose reduction, n (%)	32 (43)
venetoclax, n (%)	6 (19)
median dose reduction (%)	50
azacitdine/cytarabine/decitabine, n (%)	26 (81)
median dose reduction (%)	40
Reduction in cycle duration, n (%)	51 (68)
venetoclax, n (%)	39 (76)
median reduction, weeks (range)	7 (0.5–3)
azacitidine/cytarabine/decitabine, n (%)	12 (24)
median reduction, days (range)	2 (1–6)

^1^ One patient each received (cycle × days): 142 and 1 × 28; 2 × 42 and 3 × 28; 4 ×4 2 and 2 × 28; 4 × 42 and 9 × 28; 3 × 42 and 14 × 28; 1 × 41 and 1 × 28; 1 × 42 and 1 × 28; 2 × 42 and 9 × 28; 2 × 42 and 5 × 28; 1 × 42 and 2 × 28; 1 × 42 and 1 × 28; 1 × 42 and 2 × 28; 2 × 42 and 6 × 28; 1 × 42 and 1 × 28 d; ^2^ One patient each received (cycle × combination drug): 1× azacitidine and 1× decitabine; 6× navitoclax; 1× low dose cytarabine/cladribine; 4× azacitidine and 1× low dose cytarabine; 2× azacitidine and 1× low dose cytarabine/cladribine; 5× azacitidine and 1× navitoclax; 6× azacitidine/cusatuzumab and 2× low dose cytarabine/cladribine; 4× low dose cytarabine and 12× azacitidine; 3× azacitidine and 1× low dose cytarabine/cladribine; 3× low dose cytarabine/cladribine and 5× azacitidine; 1× azacitidine and 1× low dose cytarabine/cladribine/gilteritinib; 8× azacitidine and 4× low dose cytarabine; 1× azacitidine and 2× decitabine; 6× azacitidine and 1× low dose cytarabine/cladribine; 2× low dose cytarabine/cladribine and 2× decitabine; ^3^ One patient each received (cycles × dose): 2 × 400 mg and 1 × 100 mg; 1 × 400 mg and 2 × 100 mg; 4 × 100 mg and 2 × 400 mg; 1 × 100 mg and 19 × 400 mg; 6 × 400 mg and 2 × 100 mg; 2 × 100 mg, 12 × 200 mg and 3 × 400 mg; 5 × 100 mg and 11 × 200 mg; 1 × 100 mg and 14 × 400 mg; 6 × 100 mg and 2 × 200 mg; 3 × 200 mg; 2 × 400 mg and 13 × 200 mg; 6 × 100 mg, 2 × 150 mg, 2 × 200 mg and 1 × 400 mg; 2 × 100 mg and 3 × 400 mg; 2 × 400 mg, 2 × 300 mg and 10 × 200 mg; 4 × 100 mg and 5 × 200 mg; 2 × 100 mg and 6 × 400 mg; 2 × 70 mg; 4 × 70 mg; 5 × 400 mg and 4 × 100 mg; 3 × 70 mg.

**Table 3:** Outcomes of venetoclax therapy.

Outcome	All Patients (n = 75)
Best response	
complete remission, n (%)	49 (65)
partial remission, n (%)	10 (13)
stable disease, n (%)	14 (19)
unknown, n (%)	2 (3)
Number of cycles to best response, median (range)	1 (1–3)
1, n (%)	62 (83)
2, n (%)	12 (16)
3, n (%)	1 (1)
Progression	
no, n (%)	25 (33)
yes, n (%)	50 (67)
Time to progression after start of venetoclax, months, median (range)	5 (0.5–23)
1, n (%)	11 (22)
2, n (%)	21 (42)
3, n (%)	28 (56)
5, n (%)	35 (70)
8, n (%)	41 (82)
15, n (%)	49 (98)
23, n (%)	50 (100)
Death	
no, n (%)	30 (40)
yes, n (%)	45 (60)
Time until death after start of venetoclax, months, median (range)	7.5 (0.5–25.5)
1, n (%)	7 (16)
2, n (%)	13 (29)
3, n (%)	19 (42)
4, n (%)	24 (53)
8, n (%)	34 (76)
15, n (%)	42 (93)
26, n (%)	45 (100)
Death due to	
progression or complications, n (%)	45 (100)
other, n (%)	0 (0)

**Table 4:** Correlation between CR status and venetoclax plasma levels and hematologic recovery after the first cycle of venetoclax.

		Total, n = 72	Platelets >100 G/L ^1^	Thrombocytopenia <100 G/L ^1^	*p*-Value (<0.05)
Complete remission	yes, n (%)	38 (53)	26 (68)	12 (32)	0.002
	no, n (%)	34 (47)	10 (29)	24 (71)	
Trough level, µg/L	median	1220	1209	1231	0.22
	min.	53	53	290	
	max.	6649	6649	4169	
Peak level, µg/L	median	2117	2091	2222	0.93
	min.	419	439	419	
	max.	6607	6607	4512	
		Total, n = 72	Neutrophils >0.5 G/L ^1^	Neutropenia <0.5 G/L ^1^	*p*-Value (<0.05)
Complete remission	yes, n (%)	38 (53)	27 (71)	11 (29)	0.02
	no, n (%)	34 (47)	14 (41)	20 (59)	
Trough level, µg/L	median	1220	1193	1443	0.62
	min.	53	65	53	
	max.	6649	6649	3300	
Peak level, µg/L	median	2117	2035	2534	0.76
	min.	419	439	419	
	max.	6607	5366	6607	
		Total, n = 72	Neutrophils >1.0 G/L ^1^	Neutropenia <1.0 G/L ^1^	*p*-Value (<0.05)
Complete remission	yes, n (%)	38 (53)	23 (61)	15 (39)	0.02
	no, n (%)	34 (47)	11 (32)	23 (68)	
Trough level, µg/L	median	1220	1151	1340	0.52
	min.	53	65	53	
	max.	6649	6649	3516	
Peak level, µg/L	median	2117	1972	2328	0.43
	min.	419	439	419	
	max.	6607	4905	6607	

^1^ Measured after the first cycle of venetoclax (before the start of the second cycle).

**Table 5:** Correlation between CR status or venetoclax trough and peak levels and neutrophil and platelet dynamics before and after the first treatment cycle.

	Platelets >100 ^1^, >100 ^2^ G/L	Platelets >100 ^1^, <100 ^2^ G/L	*p*-Value (<0.05)	Platelet Recovery (>100 G/L)	No Platelet Recovery (<100 G/L)	*p*-Value (<0.05)
n = 72 CR ^3^, n (%) no CR ^3^, n (%)	13 (34) 5 (15)	2 (5) 4 (12)	0.15	13 (34) 5 (15)	10 (26) 20 (59)	0.02
Trough level, µg/L median	1586	1663	0.57	1133	1206.2	0.44
Peak level, µg/L median	1067	2483	0.84	2126	2222	0.66
	Neutrophils >1.0 ^1^, >1.0 ^2^ G/L	Neutrophils >1.0 ^1^, <1.0 ^2^ G/L	*p*-Value (<0.05)	Neutrophil recovery (>1.0 G/L)	No neutrophil recovery (<1.0 G/L)	*p*-Value (<0.05)
n = 72 CR ^3^, n (%) no CR ^3^, n (%)	14 (37) 5 (15)	4 (11) 5 (15)	0.21	9 (24) 6 (17)	11 (29) 18 (53)	0.21
Trough level, µg/L median	1030	1000	0.72	1193	1514	0.46
Peak level, µg/L median	1326	2222	0.19	2099	2534	0.81
	Neutrophils >0.5 ^1^, >0.5 ^2^ G/L	Neutrophils >0.5 ^1^, <0.5 ^2^ G/L	*p*-Value (<0.05)	Neutrophil recovery (>0.5 G/L)	No neutrophil recovery (<0.5 G/L)	*p*-Value (<0.05)
n = 72 CR ^3^, n (%) no CR ^3^, n (%)	18 (47) 11 (32)	4 (11) 7 (21)	0.17	9 (24) 3 (9)	7 (18) 13 (38)	0.07
Trough level, µg/L median	1139	1599	0.72	1229	1443	0.14
Peak level, µg/L median	1873	2755	0.09	2126	2534	0.12

^1^ Measured at the start of the first cycle of venetoclax. ^2^ Measured after the first cycle of venetoclax (before the start of the second cycle). ^3^ Complete remission.

**Table 6:** Correlation between venetoclax treatment duration and CR status or hematologic recovery after the first cycle of venetoclax.

n = 72, Venetoclax Duration	CR ^1^	No CR ^1^	*p*-Value
≤14 days, n (%) >14 days, n (%)	10 (56) 29 (54)	8 (44) 25 (46)	>0.99
n = 72, Venetoclax duration	Plateletes ^2^ >100 G/L	Thrombocytopenia ^2^ <100 G/L	*p*-Value (0.05)
≤14 days, n (%) >14 days, n (%)	9 (50) 26 (48)	9 (50) 28 (52)	>0.99
n = 72, Venetoclax duration	Neutrophils ^2^ >1.0 G/L	Neutropenia ^2^ <1.0 G/L	*p*-Value (<0.05)
≤14 days, n (%) >14 days, n (%)	8 (44) 26 (48)	10 (56) 28 (52)	>0.99
n = 72, Venetoclax duration	Neutrophils ^2^ >0.5 G/L	Neutropenia ^2^ <0.5 G/L	*p*-Value (<0.05)
≤14 days, n (%) >14 days, n (%)	11 (61) 30 (56)	7 (39) 24 (44)	0.79

^1^ Complete remission. ^2^ Measured after the first cycle of venetoclax (before the start of the second cycle).

**Table 7:** Correlation between venetoclax treatment duration and changes in neutrophil and platelet levels from before to after the first cycle of venetoclax.

n = 72, Venetoclax Duration	Platelets >100 ^1^, >100 ^2^ G/L	Platelets >100 ^1^, <100 ^2^ G/L	*p*-Value (<0.05)	Platelet Recovery (>100 G/L)	No Platelet Recovery (<100 G/L)	*p*-Value
≤14 days, n (%)	7 (39)	1 (6)	0.62	2 (11)	8 (44)	0.46
>14 days, n (%)	11 (20)	5 (9)		15 (28)	23 (43)	
n = 72, Venetoclax duration	Neutrophils >1.0 ^1^, >1.0 ^2^ G/L	Neutrophils >1.0 ^1^, <1.0 ^2^ G/L	*p*-Value (<0.05)	Neutrophil recovery (>1.0 G/L)	No neutrophil recovery (<1.0 G/L)	*p*-Value (<0.05)
≤14 days, n (%)	6 (33)	3 (17)	>0.99	2 (11)	7 (39)	0.46
>14 days, n (%)	13 (24)	7 (13)		13 (24)	21 (39)	
n = 72, Venetoclax duration	Neutrophils >0.5 ^1^, >0.5 ^2^ G/L	Neutrophils >0.5 ^1^, <0.5 ^2^ G/L	*p*-Value (<0.05)	Neutrophil recovery (>0.5 G/L)	No neutrophil recovery (<0.5 G/L)	*p*-Value (<0.05)
≤14 days, n (%)	9 (50)	3 (17)	>0.99	2 (11)	4 (22)	>0.99
>14 days, n (%)	20 (37)	8 (15)		10 (19)	16 (30)	

^1^ Measured at the start of the first venetoclax cycle. ^2^ Measured before the start of the second venetoclax cycle.

**Table 8:** Results of multiple linear regression analyses.

Dependent Variables	Independent Variables	Odds Ratio	95% CI
Complete remission (CR) ^1^	sex	0.246	0.035 to 1.292
	age > median	2.080	0.491 to 10.12
	adverse risk (ELN)	0.063	0.004 to 0.572
	de novo AML	1.003	0.215 to 4.472
	venetoclax dose > 100 mg	0.177	0.006 to 2.281
	trough levels > median	0.262	0.019 to 2.639
	peak levels > median	3.432	0.413 to 37.88
Platelets < 100 G/L ^1^	sex	5.155	0.486 to 86.37
	age > median	0.268	0.024 to 1.866
	adverse risk (ELN)	67.48	1.657 to 19139
	de novo AML	1.125	0.124 to 12.81
	venetoclax dose > 100 mg	3.524	0.058 to 223.2
	CR after the first cycle of venetoclax	0.131	0.011 to 0.890
	trough levels > median	46.02	1.335 to 5179
	peak levels > median	0.128	0.005 to 2.344
	platelets < 100 before venetoclax	43.30	2.570 to 2082
Neutrophils < 1.0 G/L ^1^	sex	0.437	0.072 to 2.478
	age > median	0.229	0.035 to 1.118
	adverse risk (ELN)	3.538	0.393 to 48.26
	de novo AML	0.834	0.160 to 4.416
	venetoclax dose > 100 mg	2.601	0.226 to 73.98
	CR after the first cycle of venetoclax	0.517	0.089 to 2.808
	trough levels > median	2.023	0.238 to 23.96
	peak levels > median	1.048	0.095 to 8.788
	neutrophils <1.0 before venetoclax	2.999	0.548 to 20.17
Neutrophils < 0.5 G/L ^1^	sex	0.506	0.086 to 2.762
	age > median	0.205	0.031 to 1.011
	adverse risk (ELN)	4.672	0.449 to 79.67
	de novo AML	2.821	0.565 to 17.33
	venetoclax dose > 100 mg	3.555	0.217 to 122.6
	CR after the first cycle of venetoclax	0.275	0.042 to 1.423
	trough levels > median	0.652	0.059 to 6.062
	peak levels > median	2.923	0.364 to 30.64
	neutrophils <0.5 before venetoclax	4.212	0.938 to 23.49
Trough levels > median ^1^	sex	0.368	0.119 to 1.074
	age > median	1.500	0.513 to 4.459
	venetoclax dose > 100 mg	0.380	0.069 to 1.743
Peak levels > median ^1^	sex	0.513	0.145 to 1.739
	age > median	1.326	0.382 to 4.701
	venetoclax dose > 100 mg	3.439	0.379 to 76.31

^1^ Measured after the first cycle of venetoclax.

## Data Availability

No data supporting the reported results are deposited elsewhere.

## Supplementary Materials

The following supporting information can be downloaded at: https://www.mdpi.com/article/10.3390/cancers17071138/s1, Table S1: Summary of MS/MS parameters for venetoclax and its corresponding internal standard; Table S2: Non-aggregated clinical data of all patients; Table S3: Treatment lines before and after venetoclax-based regimens; Table S4: Trough and peak levels of venetoclax; Table S5: Hematologic toxicity observed during the first and second cycle of venetoclax; Table S6: Association of venetoclax levels with hematologic regeneration and rate of CR; Table S7: Association of venetoclax levels with sex; Table S8: Association of venetoclax levels with dose of venetoclax; Table S9: Association of venetoclax levels with patient age at first diagnosis; Table S10: Association of venetoclax levels with the combination agent.
