# Supplementary material for: Impact of Venetoclax Treatment Schedule on Hematologic Recovery and Treatment Response in AML Patients Unfit for Intensive Chemotherapy
Source: Cancers (Basel). 2025 Mar 28;17(7):1138. doi: 10.3390/cancers17071138 (PMC11987944; doi:10.3390/cancers17071138)
Supplement: Supplementary file 1 [file cancers-17-01138-s001.zip › cancers-3461009-supplementary.pdf]

Supplemental Material

Supplemental Table S1: Summary of MS/MS parameters for venetoclax and its corresponding internal standard.

Name	Parent Ion [m/z]	Product Ion	Collision energy
venetoclax quantifier	868.1	321.2	-37
venetoclax qualifier	868.1	636.3	-27
[$^2H_7$]-venetoclax quantifier	875.2	321.2	-38
[$^2H_7$]-venetoclax qualifier	875.2	643.3	-28

Supplemental Table S2: Non-aggregated clinical data of all patients.

Patient	Patient characteristics at first diagnosis						
	Patient information		Lab				
	Sex (male or female)	Age at first diagnosis	Leukocytes (G/l)	Hb (G/l)	Platelets (G/l)	LDH (G/l)	Blasts (%)
1	m	75	8.1	83	19	453	42
2	f	74	27.4	78	108	746	30
3	m	59	3	82	9	1022	30
4	f	43	9.1	97	30	679	52
5	m	73	2.6	96	286	156	14
6	m	64	1.2	76	186	454	2
7	f	67	10.4	82	104	498	80
8	m	71	7.4	75	329	800	14.5
9	f	66	1.6	97	311	490	4
10	m	68	3.2	101	38	433	40
11	f	67	4	93	46	271	88
12	m	72	2.9	132	72	477	1
13	m	66	5.4	109	122	270	5
14	f	81	1.3	62	90	207	-
15	f	70	4.3	90	31	488	3
16	f	83	6.5	84	140	249	62
17	m	71	120	129	54	583	13
18	m	62	2.2	82	341	287	1
19	m	52	40	92	98	715	92
20	m	75	6	79	336	845	10
21	f	75	174	122	80	1091	85
22	m	61	2.5	119	68	243	0
23	m	38	7.7	115	29	326	10
24	m	78	1	82	27	245	0
25	f	78	0.6	59	70	255	38
26	m	75	5.7	63	25	353	41
27	f	60	0.6	72	85	245	2
28	m	33	12.6	125	92	902	44
29	m	73	5.7	70	108	569	78
30	f	58	0.3	73	83	194	5
31	m	77	2.9	106	64	369	0
32	m	77	9.6	88	80	313	18
33	f	64	5.6	72	21	503	42
34	m	65	2.4	67	99	208	2
35	m	62	40.7	96	65	1664	47
36	F	75	85.5	96	108	1711	18
37	f	49	71.4	88	38	1103	90
38	m	53	5.2	101	119	210	40
39	m	57	2.7	117	4	350	79
40	m	71	1.3	69	56	287	6.5
41	f	69	13.6	69	112	401	37.5
42	m	30	4.5	123	8	6553	60
43	f	65	15.4	91	5	215	22
44	m	53	1.2	90	87	306	3
45	m	54	10.1	120	133	281	7

46	f	78	8	96	39	-	63
47	f	69	35.5	77	48	648	42
48	f	83	1.5	116	76	408	0
49	m	73	2.2	97	77	392	0
50	f	78	28	82	87	490	11
51	m	60	0.2	80	37	215	7
52	f	81	42	95	296	442	15
53	f	63	41.1	83	13	762	18
54	m	33	88	116	46	882	82
55	m	69	2	103	246	218	0
56	f	63	3.2	95	22	220	31
57	f	47	1.6	94	33	220	0
58	f	71	1.7	72	231	487	33
59	m	76	74.8	68	15	1177	55
60	m	71	4.1	113	15	977	35
61	m	85	1.4	96	40	210	0
62	f	76	2.7	106	70	197	0.5
63	m	78	39	118	85	831	45
64	f	75	18.1	83	50	1549	57
65	f	72	4.9	74	156	210	20
66	m	77	1.6	107	62	342	5
67	m	82	1.2	71	40	175	2
68	m	75	2.6	46	102	215	2
69	m	79	2.5	105	64	194	12
70	m	44	107	37	18	885	1.3
71	f	63	1	65	49	172	2
72	m	70	56	73	23	1022	90
73	f	57	169	98	61	1640	77
74	m	65	76.2	95	30	1806	70
75	m	77	1.5	82	38	282	1

Patient	**Patient characteristics at first diagnosis**					
	Lab			ELN risk category	Molecular diagnostics	Cytogenetics
	AML-FAB subtype (M0 to M7)	Primary or secondary AML	Bone marrow infiltration with blasts (%)	Favorable, intermediate or adverse	Mutations in…	Karyotype
1	-	secondary	40	adverse	TP53	complex and monosomal
2	-	secondary	20	adverse	RUNX1, SH2B3, SRSF2, TET2	single anomalies
3	M0	primary	30	adverse	TP53	complex and monosomal
4	-	secondary	90	adverse	NOTCH1	complex and monosomal
5	M1	primary	90	adverse	DNMT3A, IDH1, IDH2, TP53	complex and monosomal
6	-	secondary	30	adverse	-	single anomalies

7	M2	primary	90	adverse	ASXL1, TET2	normal
8	-	secondary	30	adverse	GATA2, RB1, SF3B1	single anomalies
9	-	secondary	25	adverse	GATA2, PTPN11, SF3B1, SH2B3, TET2	single anomalies
10	-	secondary	30	adverse	BCOR, NRAS, RUNX1, U2AF1	single anomalies
11	M0	primary	90	adverse	BCOR, DNMT3A, FLT3, IDH1, IKZF1, PTPN11	single anomalies
12	M5	primary	90	adverse	ASXL1, EZH2, FLT3, JAK2, NPM1, TET2	single anomalies
13	M4	primary	20	adverse	DNMT3A, ETV6, NRAS, U2AF1	normal
14	-	secondary	30	adverse	BCOR, DNMT3A, IDH2, JAK2, SF3B1, TET2	single anomalies
15	-	secondary	60	adverse	TP53	complex and monosomal
16	M0	primary	90	intermediate	JAK3, NOTCH1	single anomalies
17	-	secondary	80	favorable	IDH2, NPM1, SRSF2	single anomalies
18	-	secondary	20	adverse	ASXL1, EZH2, IDH1	single anomalies
19	M1	primary	90	adverse	FLT3, IDH1, NPM1, RUNX1	normal
20	-	secondary	20	adverse	ETV6, GATA2, KRAS, NF1, SF3B1, WT1	single anomalies
21	M2	primary	90	adverse	CBL, SF3B1, TET2	normal
22	-	secondary	20	adverse	ASXL1, RUNX1, SRSF2, STAG2, TET2	normal
23	-	secondary	30	adverse	TP53	normal
24	M0	primary	60	adverse	ASXL1, CBL, CTNNA1, SETBP1, SUZ12,	complex
25	M1	primary	90	adverse	BCOR, DNMT3A, IDH2, RUNX1, SRSF2	normal
26	M4	primary	80	favorable	NPM1, NRAS, SMC1A	normal
27	M1	primary	90	adverse	BCOR, DNMT3A, IDH2	normal
28	M2	primary	90	intermediate	DNMT3A, FLT3, NPM1, NRAS	normal
29	M4	primary	95	adverse	DNMT3A, IDH1, NPM1, SF3B1, ZRSR2	complex

30	-	secondary	20	adverse	JAK2, TP53	complex
31	-	secondary	30	adverse	ASXL1, IDH2	normal
32	-	secondary	70	adverse	CBL, RUNX1, SRSF2	single anomalies
33	-	secondary	40	adverse	TP53	complex and monosomal
34	M2	primary	25	adverse	TP53	normal
35	M4	primary	50	adverse	ASXL1, EZH2, KRAS, NRAS, SRSF2, STAG2	single anomalies
36	M4	primary	50	intermediate	DNMT3A, FLT3, NPM1, SUZ12	normal
37	-	secondary	60	intermediate	FLT3, NF1, ZBTB7A	normal
38	M4	primary	55	adverse	IDH1, NF1, RUNX1, TP53	complex and monosomal
39	M1	primary	90	adverse	ASXL2, IDH2, PTPN11, SMC1A, SRSF2	single anomalies
40	-	secondary	80	adverse	IDH2, RUNX1	single anomalies
41	-	secondary	70	adverse	BCOR, DNMT3A, IDH2, NRAS	single anomalies
42	M1	primary	60	adverse	EZH2	single anomalies
43	-	secondary	25	adverse	PTPN11, RUNX1, SF3B1, WT1	single anomalies
44	-	secondary	90	adverse	-	complex and monosomal
45	M4	primary	30	favorable	IDH2, NPM1, PHF6, SF3B1	normal
46	M2	primary	80	adverse	RUNX1, SH2B3, TET2	single anomalies
47	M1	primary	90	favorable	CTCF, DNMT3A, IDH2, NPM1	normal
48	M2	primary	20	adverse	SRSF2	normal
49	-	secondary	25	adverse	ASXL1, RUNX1	single anomalies
50	M1	primary	95	intermediate	DNMT3A, FLT3, NPM1, PTPN11, TET2	normal
51	M0	primary	40	adverse	CUX1, DDX41, SRSF2	normal
52	-	secondary	40	adverse	ASXL1, EZH2, GATA2, NRAS, TET2	normal
53	M2	primary	60	intermediate	CEBPA	normal
54	M4	primary	95	favorable	-	single anomalies
55	M0	primary	60	adverse	DNMT3A	single anomalies

56	M1	primary	80	adverse	ASXL1, FLT3, SRSF2, STAG2	normal
57	-	secondary	20	intermediate	WT1	normal
58	M0	primary	85	adverse	PTPN11, TP53	complex
59	M2	primary	60	adverse	ASXL1, GATA2, NF1, NRAS, RUNX1, SRSF2, STAG2	normal
60	-	secondary	90	adverse	TP53	complex and monosomal
61	-	secondary	20	adverse	ASXL1, RUNX1, STAG2	normal
62	-	secondary	20	adverse	DDX41, SF3B1	normal
63	M4	primary	95	favorable	ASXL1, DNMT3A, IDH2, NPM1, NRAS, TET2	normal
64	M1	primary	80	adverse	CUX1, KRAS, TET2, TP53, U2AF1	-
65	M2	primary	95	favorable	NPM1, TET2	normal
66	-	secondary	30	adverse	ASXL1, SRSF2, STAG2, TET2	single anomalies
67	-	secondary	70	adverse	ASXL1, GATA2, NRAS	single anomalies
68	-	secondary	20	adverse	U2AF1	single anomalies
69	M2	primary	50	adverse	KRAS, TET2	single anomalies
70	M5	primary	90	intermediate	DNMT3A, FLT3, NPM1	normal
71	M0	primary	20	adverse	TET2, TP53	complex and monosomal
72	M5	primary	70	adverse	DNMT3A, RUNX1, SRSF2	single anomalies
73	M1	primary	80	intermediate	FLT3, NPM1, TET2, WT1	normal
74	-	secondary	90	favorable	ASXL1, NPM1, SRSF2, TET2	single anomalies
75	M2	secondary	30	adverse	DDX41, SRSF2	normal

Patient	**Treatment line(s) before venetoclax (if any)**			**Treatment line(s) after venetoclax (if any)**		
	Type	Outcome		Type	Outcome	
		Best response (CR, PR or SD)	Relapse/Progression after … months (TTNT)		Best response (CR, PR or SD)	Relapse/Progression after … months (TTNT)
1	chemotherapy	CR	8	-	-	-
2	-	-	-	-	-	-

3	chemotherapy and allogeneic SCT	CR	10.5	-	-	-
4	chemotherapy and allogeneic SCT	CR	55	-	-	-
5	-	-	-	immunotherapy	-	-
6	chemotherapy	CR	79	immunotherapy	SD	-
7	chemotherapy and autologous SCT	CR	9	-	-	-
8	chemotherapy	CR	19	-	-	-
9	chemotherapy and allogeneic SCT	CR	8	-	-	-
10	chemotherapy	CR	14	-	-	-
11	chemotherapy and autologous SCT	CR	7	-	-	-
12	-	-	-	-	-	-
13	chemotherapy and autologous SCT	CR	12	chemotherapy and allogeneic SCT	CR	3
14	-	-	-	-	-	-
15	-	-	-	-	-	-
16	-	-	-	-	-	-
17	chemotherapy	CR	47	-	-	-
18	-	-	-	-	-	-
19	chemotherapy and autologous SCT	CR	18	chemotherapy	-	-
20	-	-	-	-	-	-
21	-	-	-	-	-	-
22	-	-	-	chemotherapy and allogeneic SCT	CR	-
23	-	-	-	-	-	-
24	-	-	-	-	-	-
25	-	-	-	immunotherapy	-	1
26	-	-	-	-	-	-
27	chemotherapy and allogeneic SCT and autologous SCT	CR	34	chemotherapy and allogeneic SCT	CR	1
28	chemotherapy and autologous SCT	CR	14	chemotherapy and allogeneic SCT	CR	-
29	chemotherapy and autologous SCT	CR	6.5	-	-	-

30	-	-	-	chemotherapy and allogeneic SCT	CR	3
31	chemotherapy	CR	46	-	-	-
32	-	--	-	-	-	-
33	chemotherapy	PR	1	-	-	-
34	chemotherapy	CR	16.5	chemotherapy and immunotherapy	PR	3
35	-	-	-	-	-	-
36	-	-	-	-	-	-
37	chemotherapy	CR	1.5	immunotherapy	PR	-
38	chemotherapy	PR	1	chemotherapy and allogeneic SCT	CR	-
39	chemotherapy	CR	4	-	-	-
40	-	-	-	-	-	-
41	-	-	-	-	-	-
42	chemotherapy and allogeneic SCT	CR	15	chemotherapy and allogeneic SCT	CR	-
43	chemotherapy	CR	3	chemotherapy	SD	1
44	-	-	-	chemotherapy and allogeneic SCT and autologous SCT	CR	12
45	chemotherapy	CR	3.5	chemotherapy and autologous SCT	CR	-
46	-	-	-	-	-	-
47	-	-	--	-	-	-
48	chemotherapy	CR	40	-	-	-
49	-	-	-	-	-	-
50	-	-	--	-	-	-
51	chemotherapy and autologous SCT	CR	18	-	-	-
52	-	-	-	-	-	-
53	-	-	--	chemotherapy and autologous SCT	CR	-
54	Chemotherapy, allogeneic SCT and autologous SCT	CR	52	chemotherapy and allogeneic SCT	CR	-
55	chemotherapy and allogeneic SCT	CR	7	-	-	-
56	chemotherapy	CR	6	-	-	-
57	-	-	-	-	-	-
58	-	-	-	-	-	-

59	-	-	-	chemotherapy	SD	-
60	-	-	-	-	-	-
61	-	-	-	-	-	-
62	-	-	-	-	-	-
63	-	-	-	-	-	-
64	-	-	-	-	-	-
65	-	-	-	-	-	-
66	-	-	-	-	-	-
67	-	-	-	-	-	-
68	-	-	-	-	-	-
69	-	-	-	-	-	-
70	Chemotherapy, allogeneic SCT and autologous SCT	CR	47	-	-	-
71	-	-	-	-	-	-
72	chemotherapy	CR	38	chemotherapy and allogeneic SCT	CR	-
73	-	-	-	-	-	-
74	chemotherapy	CR	3	-	-	-
75	-	-	-	-	-	-

Patient	**Treatment with venetoclax**				
	Cycles		Dosis (in mg)	Combination with	Relevant co-medication (CYP3A…)
	Number	Duration (days)			
1	3	28	400 and 100	vidaza	fluconazol, isavuconazol
2	2	42 and 28	100	vidaza and decitabine	posaconazol
3	1	28	400	decitabine	-
4	6	28	400	navitoclax	-
5	5	42 and 28	100	vidaza and cytarabin	posaconazol
6	11	28	400	decitabine	-
7	1	28	400	vidaza	-
8	1	28	100	cytarabin/cladribin	posaconazol
9	3	28	100	vidaza and cytarabin/cladribin	posaconazol, isavuconazol
10	3	28	400	vidaza	posaconazol
11	2	42	400	vidaza	-
12	6	28	100 and 400	vidaza	posaconazol
13	6	42 and 28	100	vidaza and navitoclax	posaconazol, voriconazol
14	2	28	100	vidaza	posaconazol
15	3	42	100	vidaza	posaconazol
16	18	42 and 28	100 and 400	vidaza	posaconazol
17	5	28	100	vidaza	posaconazol
18	5	42	100	vidaza	posaconazol
19	1	28	100	decitabine	posaconazol
20	8	28	400 and 100	vidaza/cusatuzumab and cytarabin/cladribin	posaconazol

21	4	42	100	vidaza	posaconazol
22	1	28	400	vidaza	-
23	2	28	100	vidaza	posaconazol
24	1	42	100	vidaza	posaconazol
25	13	42 and 28	100	vidaza	posaconazol
26	17	42 and 28	100, 200 and 400	vidaza	posaconazol and isavuconazol
27	16	28	100 and 200	cytarabin, vidaza	posaconazol
28	1	42	400	vidaza	-
29	2	42 and 28	100	vidaza	posaconazol
30	13	28 and 42	100 and 200	vidaza	posaconazol
31	5	28	400	vidaza	-
32	1	28	100	decitabine	-
33	4	28	100	vidaza and cytarabin/cladribin	posaconazol
34	8	28	100	vidaza and cytarabin/cladribin	posaconazol
35	1	42	100	vidaza	posaconazol
36	15	42 and 28	100 and 400	vidaza	posaconazol
37	2	42 and 28	100	vidaza and cytarabin/cladribin/gilteritinib	posaconazol
38	4	42	100	vidaza	posaconazol
39	5	42	100	vidaza	posaconazol
40	12	42 and 28	100	vidaza and cytarabin	voriconazol
41	11	42 and 28	100 and 400	vidaza	posaconazol
42	7	42 and 28	100 and 200	vidaza	posaconazol
43	2	42	100	vidaza	isavuconazol
44	2	42	100	vidaza	posaconazol
45	3	42 and 28	200	vidaza and decitabine	-
46	1	42	100	vidaza	voriconazol
47	6	42	100	vidaza	posaconazol
48	8	42 and 28	100	vidaza	posaconazol
49	15	42	400 and 200	vidaza	-
50	11	28	400, 200, 150 and 100	vidaza	posaconazol
51	1	42	100	vidaza	posaconazol
52	2	42 and 28	100	vidaza	posaconazol
53	5	42 and 28	100 and 400	vidaza	posaconazol
54	14	28	400, 300, 200 and 100	vidaza	-
55	9	28	100 and 200	vidaza	isavuconazol
56	6	42 and 28	100	vidaza	posaconazol
57	15	42	100	vidaza	posaconazol
58	2	42	100	vidaza	posaconazol
59	3	42 and 28	100	vidaza	posaconazol
60	1	42	100	vidaza	posaconazol
61	8	42 and 28	100 and 400	vidaza	posaconazol

62	9	42	100	vidaza	posaconazol
63	18	42 and 28	400	vidaza	-
64	2	28	70	vidaza	posaconazol
65	6	42	100	vidaza	posaconazol
66	7	42	100	vidaza and cytarabin/cladribin	posaconazol
67	4	28	70	vidaza	posaconazol
68	9	28	400, 200 and 100	Vidaza and cytarabin/cladribin	posaconazol
69	1	42	100	vidaza	posaconazol
70	10	42	100	vidaza	posaconazol
71	3	28	70	vidaza	posaconazol
72	4	28	100	Cytarabin/cladribin and decitabin	posaconazol
73	2	42 and 28	400	vidaza	-
74	1	28	100	decitabin	posaconazol
75	11	42 and 28	100	vidaza	posaconazol

Patient	**Treatment with venetoclax**							
	Venetoclax levels				Outcome			
	Which cycle?	Which day after start of cycle?	Peak level (µg/l)	Trough level (µg/l)	best response (CR, PR or SD)	best response after … cycles?	Progression (yes or no?)	Progression after … months after start venetoclax
1	3	5	-	61	SD	1	yes	3
2	1	4	2968	1724	PR	1	yes	2
3	1	12	-	1193	CR	1	yes	1
4	1	21	-	427	CR	1	yes	3.5
		27	-	311				
	2	4	-	681				
		26	-	1329				
	3	24	-	708				
		20	-	587				
5	1	7	1653	1230	PR	1	yes	4
		14	1388	1297				
	2	8	-	3717				
		22	-	2078				
6	7	4	-	2364	CR	1	yes	23
	9	4	-	2020				
		7	-	2324				
7	1	4	4058	3301	SD	1	yes	1
		12	4039	3300				
8	1	4	1997	1601	SD	1	yes	0.5
9	3	5	809	114	SD	1	yes	3
		19	-	200				
10	3	14	-	1085	CR	1	yes	3
11	2	9	-	1765	CR	1	yes	3
		13	3343	2280				
12	1	4	-	551	CR	1	yes	4
		5	-	689				

13	1	5	3495	1982	CR	2	yes	7.5
	2	7	3763	2926				
		15	3436	2574				
	3	8	2340	1607				
	4	5	1992	1621				
14	1	4	3413	1592	SD	1	yes	2
	2	7	3680	1989				
15	1	6	2117	780	PR	2	yes	2.5
	2	22	-	1856				
16	1	7	3911	2739	CR	1	no	20
		14	5408	4653				
	2	5	-	1400				
	3	4	-	1653				
	4	5	-	1131				
17	1	4	2553	1280	CR	1	yes	5.5
		15	-	2553				
	3	12	-	3206				
18	1	6	2817	2497	PR	1	yes	5
19	1	6	777	715	CR	1	yes	1
		17	62	41				
20	7	4	801	550	CR	2	yes	1
21	1	7	-	889	CR	2	no	28.5
	2	7	-	1906				
	3	4	2272	1335				
	4	5	1079	924				
22	1	5	-	330	CR	1	no	27
		15	-	699				
23	1	4	-	681	SD	1	yes	2
		5	-	1033				
		14	-	1407				
	2	5	-	748				
		6	-	658				
		15	-	1371				
24	1	6	3066	1559	PR	1	yes	4
25	1	5	1703	1133	CR	1	yes	15
	4	8	-	723				
	5	9	-	571				
	6	15	-	830				
	7	15	-	727				
	9	15	-	446				
26	1	5	2126	932	CR	1	no	22
	3	5	-	1489				
		10	-	865				
	6	8	-	499				
	8	15	-	501				
	9	7	-	492				
27	1	7	-	257	CR	1	no	20.5
	2	21	-	1779				
	3	8	-	503				
	4	8	-	6489				

	7	5	-	359				
28	1	5	-	1209	CR	1	no	25
29	1	4	2601	707	PR	1	yes	1
		9	4186	2554				
	2	5	1398	1169				
30	1	4	606	53	CR	2	no	28.5
	3	4	384	275				
	9	5	-	320				
31	1	11	-	290	SD	1	yes	5
	5	4	-	610				
32	1	10	-	1868	PR	1	yes	1
33	1	4	1200	684	SD	1	yes	3.5
		5	-	795				
	2	5	2882	1776				
	3	6	-	1886				
34	1	14	506	140	CR	1	yes	11
35	1	7	3398	2090	CR	1	yes	2
36	1	7	4905	3349	CR	1	no	16.5
	2	5	-	2664				
		8	-	2447				
		15	-	1539				
37	1	5	547	123	CR	2	yes	4.5
		13	-	450				
	2	5	422	140				
38	1	5	2072	1151	CR	1	no	12
	3	10	-	885				
39	1	5	-	1876	CR	1	no	12
		13	-	4518				
	2	5	-	2231				
		15	-	2087				
	3	5	-	1460				
	4	5	-	2947				
40	1	6	2865	1416	CR	1	yes	8
	2	5	-	573				
	4	10	-	899				
	9	7	-	1031				
41	1	6	2328	1242	CR	1	no	13
	4	5	-	949				
42	1	5	-	1908	CR	1	yes	6.5
	3	6	-	1538				
		12	-	1841				
	6	7	-	209				
		13	-	448				
43	1	6	779	200	SD	1	yes	2
		7	-	435				
44	1	7	2984	1553	SD	1	no	14
		14	2084	1793				
	2	6	2214	1151				

45	3	5	-	718	CR	1	no	6.5
		8	-	1389				
46	1	5	-	770	-	1	yes	0.5
47	1	5	1154	716	CR	1	no	5
	4	5	-	920				
		8	-	1405				
	5	5	-	1683				
48	1	5	4512	2483	PR	3	no	7.5
		9	-	5855				
	5	4	-	200				
		5	-	169				
49	1	5	6607	2425	CR	1	no	16
	2	5	9091	5172				
	9	5	-	1484				
50	1	21	439	404	CR	1	no	12
	2	6	1591	547				
51	1	5	2748	1470	SD	1	yes	1
52	1	7	614	65	CR	1	yes	1.5
	2	6	3367	2215				
53	1	8	2507	1663	CR	1	yes	13.5
	2	8	-	1716				
	3	5	-	353				
	4	15	-	961				
	5	18	-	708				
54	9	5	-	572	CR	2	no	27.5
		12	-	385				
55	6	5	-	170	CR	1	no	9.5
56	5	5	-	1229	CR	1	yes	10
	6	5	-	879				
57	1	8	-	2177	CR	1	no	17.5
		12	-	1661				
		15	-	2179				
		19	-	1779				
	2	5	-	2534				
		14	-	3390				
	3	5	-	1096				
	4	12	-	2152				
	5	12	-	6223				
58	1	5	1962	824	SD	1	yes	2.5
	2	7	3214	1614				
59	1	7	1873	1139	CR	1	yes	2
60	1	6	3585	2181	-	-	yes	0.5
61					CR	1	no	9
62	1	5	-	2375	CR	1	no	9.5
		10	-	7562				
		17	-	6649				
	4	5	-	3632				
	5	4	-	3556				

	6	5	-	3044				
	9	5	-	1948				
63	1	6	2206	921	CR	2	no	17.5
64	1	8	914	637	CR	2	yes	2
	2	7	-	1132				
		8	-	1347				
65	6	5	-	1917	CR	1	yes	9
66	1	5	-	2350	CR	2	yes	6.5
		15	-	1072				
	6	5	-	2385				
67	1	10	1948	1264	CR	2	no	4.5
	2	17	-	1883				
	3	5	-	2090				
68	8	5	-	1947	CR	1	yes	9
		8	-	3152				
	9	9	-	3168				
69	1	6	1024	471	PR	1	yes	1
70	1	8	-	3563	CR	1	no	11.5
71	3	11	-	1554	PR	1	yes	3
72	2	5	-	732	CR	2	no	19.5
		12	-	352				
	4	5	-	709				
73	2	7	2662	876	SD	1	yes	2
74	1	7	842	482	SD	1	yes	1
75	1	7	-	1036	CR	2	yes	14
		8	2091	1116				
	2	10	-	2967				

Patient	**Treatment with venetoclax**								
	Outcome								
	Cytopenia								
	At start of first venetoclax cycle			During first cycle of venetoclax			After first cycle of venetoclax (before starting next cycle)		
	Neutrophils <1.0 G/l	Neutrophils <0.5 G/l	Platelets <100 G/l	Neutrophils <1.0 G/l	Neutrophils <0.5 G/l	Platelets <100 G/l	Neutrophils <1.0 G/l	Neutrophils <0.5 G/l	Platelets <100 G/l
1	yes	yes	yes	yes	yes	yes	yes	yes	yes
2	no	no	no	no	no	yes	no	no	yes
3	yes	yes	yes	yes	yes	yes	no	no	yes
4	no	no	no	yes	no	yes	yes	no	yes
5	yes	no	no	yes	yes	yes	no	no	no
6	yes	yes	no	yes	yes	yes	yes	yes	yes
7	no	no	yes	yes	yes	yes	yes	yes	yes
8	yes	no	no	yes	yes	yes	yes	no	yes
9	-	-	-	-	-	-	-	-	-
10	yes	no	yes	yes	yes	yes	yes	no	yes
11	yes	yes	yes	yes	yes	yes	yes	yes	yes
12	yes	no	yes	yes	yes	yes	no	no	no
13	yes	yes	yes	yes	yes	yes	yes	yes	yes

14	yes	yes	yes	yes	yes	yes	no	no	yes
15	no	no	yes	yes	yes	yes	yes	yes	yes
16	yes	yes	yes	yes	yes	yes	no	no	no
17	yes	yes	yes	yes	yes	yes	yes	yes	no
18	yes	yes	no	yes	yes	yes	yes	yes	no
19	yes	yes	yes	yes	yes	yes	yes	yes	yes
20	no	no	no	yes	yes	yes	yes	yes	yes
21	no	no	yes	yes	yes	yes	no	no	no
22	no	no	yes	yes	yes	yes	no	no	yes
23	no	no	yes	yes	yes	yes	yes	yes	yes
24	yes	yes	yes	yes	yes	yes	yes	yes	yes
25	yes	yes	yes	yes	yes	yes	no	no	no
26	yes	yes	yes	yes	yes	yes	no	no	no
27	no	no	no	yes	yes	yes	no	no	no
28	no	no	no	no	no	no	no	no	no
29	yes	no	yes	yes	yes	yes	yes	yes	yes
30	yes	yes	yes	yes	yes	yes	yes	yes	no
31	yes	no	yes	yes	no	yes	yes	no	yes
32	yes	yes	no	yes	yes	yes	yes	yes	yes
33	yes	no	yes	yes	yes	yes	no	no	yes
34	no	no	no	yes	yes	yes	no	no	no
35	no	no	yes	yes	yes	yes	yes	yes	yes
36	yes	yes	yes	yes	yes	yes	no	no	no
37	no	no	no	yes	yes	no	no	no	no
38	yes	yes	yes	yes	yes	yes	no	no	no
39	no	no	no	yes	no	no	no	no	no
40	yes	yes	yes	yes	yes	yes	yes	yes	yes
41	no	no	yes	yes	yes	yes	yes	no	yes
42	no	no	no	no	no	no	no	no	no
43	no	no	yes	yes	yes	yes	no	no	yes
44	yes	yes	yes	yes	yes	yes	yes	yes	yes
45	no	no	no	no	no	no	no	no	no
46	yes	yes	yes	yes	yes	yes	-	-	-
47	no	no	yes	yes	yes	yes	yes	yes	no
48	yes	no	yes	yes	yes	yes	no	no	yes
49	yes	no	no	yes	yes	yes	yes	yes	no
50	yes	yes	yes	yes	yes	yes	no	no	no
51	yes	yes	yes	yes	yes	yes	yes	yes	yes
52	no	no	no	no	no	no	no	no	no
53	no	no	yes	yes	yes	yes	no	no	no
54	no	no	no	no	no	no	no	no	no
55	no	no	no	no	no	no	no	no	no
56	yes	yes	yes	yes	yes	yes	yes	yes	yes
57	yes	no	yes	yes	yes	yes	yes	yes	no
58	yes	yes	yes	yes	yes	yes	yes	yes	yes
59	no	no	yes	yes	yes	yes	no	no	yes
60	yes	yes	yes	yes	yes	yes	-	-	-
61	yes	yes	yes	yes	yes	yes	yes	no	no
62	no	no	no	yes	yes	no	no	no	no
63	no	no	yes	yes	yes	yes	no	no	no
64	no	no	yes	yes	yes	yes	yes	yes	yes

65	yes	yes	no	yes	yes	yes	yes	yes	no
66	yes	no	yes	yes	yes	yes	no	no	no
67	yes	yes	yes	yes	yes	yes	yes	no	yes
68	yes	yes	no	yes	yes	yes	no	no	no
69	yes	yes	yes	yes	yes	yes	yes	yes	yes
70	no	no	no	no	no	no	no	no	no
71	yes	yes	yes	yes	yes	yes	yes	yes	no
72	yes	yes	no	yes	yes	yes	no	no	no
73	yes	yes	yes	yes	yes	yes	yes	yes	yes
74	yes	yes	yes	yes	yes	yes	yes	yes	yes
75	yes	no	yes	yes	yes	yes	yes	yes	no

Patient	**Treatment with venetoclax**						
	Outcome						
	Reduction in…				Death (yes or no)	After … months after start of venetoclax	Due to progression/complications? (yes or no)
	Dose (in %)		Duration of therapy (in days)				
	Venetoclax	HMA	Venetoclax	HMA			
1	0	40	14	0	yes	3	yes
2	0	0	0	0	yes	4	yes
3	0	0	0	0	yes	4	yes
4	0	0	0	3	yes	7	yes
5	0	40	0	0	yes	5.5	yes
6	0	33	21	1	no	38.5	-
7	0	0	0	0	yes	1	yes
8	0	0	0	0	yes	1.5	yes
9	0	0	0	0	yes	12	yes
10	0	0	7	2	yes	6	yes
11	0	0	0	0	yes	3	yes
12	0	30	7	0	yes	7.5	yes
13	0	0	7	0	yes	10	yes
14	0	0	0	0	yes	2.5	yes
15	0	0	0	0	yes	3	yes
16	0	30	7	0	no	20	-
17	0	0	10	0	yes	7	yes
18	0	0	0	0	yes	11	yes
19	0	0	0	0	yes	1.5	yes
20	0	50	14	0	yes	14.5	yes
21	0	0	0	0	yes	4	yes
22	0	0	0	0	no	27	-
23	30	0	7	0	yes	3	yes
24	0	0	0	0	yes	4	yes
25	0	50	7	2	yes	20	yes
26	0	70	14	2	no	22	-
27	0	0	7	0	no	20.5	-
28	0	0	0	0	no	25	-
29	0	0	0	0	yes	1.5	yes
30	0	70	7	0	no	28.5	-
31	0	0	0	0	yes	7	yes
32	0	0	0	0	yes	1	yes

33	0	0	7	0	yes	5	yes
34	0	50	10	0	yes	25.5	yes
35	0	0	0	0	yes	2	yes
36	0	40	14	0	no	16.5	-
37	0	0	3	0	yes	4.5	yes
38	0	0	0	0	no	12	-
39	0	20	0	0	no	12	-
40	0	20	12	6	no	13	-
41	0	30	3	2	no	13	-
42	0	0	7	0	yes	12	yes
43	0	15	0	0	yes	7	yes
44	0	30	0	0	no	14	yes
45	0	25	0	0	no	6.5	-
46	0	0	0	0	yes	0.5	yes
47	0	0	0	0	no	5	-
48	0	0	7	0	no	7.5	-
49	50	0	7	0	no	16	-
50	75	0	7	2	no	12	-
51	0	0	0	0	yes	1	yes
52	0	0	6	0	yes	3.5	yes
53	0	0	0	0	yes	13.5	yes
54	75	0	20	0	no	27.5	-
55	0	0	7	0	no	9.5	-
56	30	60	7	0	yes	10	yes
57	0	50	14	2	no	17.5	-
58	0	50	0	0	yes	3	yes
59	0	25	7	0	yes	8	yes
60	0	0	0	0	yes	0.5	yes
61	0	0	7	0	no	9	-
62	0	25	14	0	no	9.5	-
63	0	50	14	0	no	17.5	-
64	0	0	18	0	yes	2	yes
65	0	40	7	0	yes	11	yes
66	0	40	14	1	no	7	-
67	0	0	0	0	no	4.6	-
68	50	50	18	1	yes	16	yes
69	0	0	0	0	yes	1	yes
70	0	0	0	0	no	11.5	-
71	0	5	10	0	no	4	-
72	0	0	7	0	no	19.5	-
73	0	0	0	0	yes	2	yes
74	0	0	0	0	yes	1	yes
75	0	0	14	0	no	14.5	-

Supplemental Table S3: Treatment lines before and after venetoclax-based regimens.

**Treatment before venetoclax**	All patients (n=75)
Yes, n (%)	32 (43)
No, n (%)	43 (57)
Chemotherapy only, n (%)	16 (50)
With autologous transplantation consolidation, n (%)	7 (22)
With allogeneic transplantation consolidation, n (%)	5 (16)
With allogeneic and autologous transplantation consolidation, n (%)	4 (13)
Best response	
complete remission, n (%)	30 (94)
partial remission, n (%)	2 (6)
stable disease, n (%)	0 (0)
Median time to next treatment, months (range)	12 (0-79)

**Treatment after venetoclax**	All patients (n=75)
Yes, n (%)	20 (27)
No, n (%)	55 (73)
Chemotherapy only, n (%)	3 (15)
With autologous transplantation consolidation, n (%)	2 (10)
With allogeneic transplantation consolidation, n (%)	10 (50)
Immunotherapy, n (%)	5 (25)
Best response	
complete remission, n (%)	12 (60)
partial remission, n (%)	2 (10)
stable disease, n (%)	3 (15)
unknown, n (%)	3 (15)
Median time to next treatment, months (range)	2 (0-12)

Supplemental Table S4: Trough and peak levels of venetoclax.

	**Trough level (µg/L)**	**Peak level (µg/L)**
Minimum value	61	419
Maximum value	3722	7849
Median	1169	2122
Percentile 10%	407	727
Percentile 25%	699	1429
Percentile 75%	1876	2993
Percentile 90%	2468	4188

Supplemental Table S5: Hematologic toxicity observed during the first and second cycle of venetoclax.

First cycle, n = 75	Platelets <100 G/L[1]	Neutrophils <1.0 G/L[1]	Neutrophils <0.5 G/L[1]
yes, n (%)	64 (85)	66 (88)	63 (84)
no, n (%)	10 (13)	8 (11)	11 (15)
unknown (%)	1 (1)	1 (1)	1 (1)
First cycle, n = 75	Platelets <100 G/L[2]	Neutrophils <1.0 G/L[2]	Neutrophils <0.5 G/L[2]
yes, n (%)	36 (48)	38 (51)	31 (41)
no, n (%)	36 (48)	34 (45)	41 (55)
unknown (%)	3 (4)	3 (4)	3 (4)

[1]Measured during the first cycle of venetoclax

[2]Measured at the time after the first cycle of venetoclax (at the start of the next cycle)

Second cycle, n = 61	Platelets <100 G/L[3]	Neutrophils <1.0 G/L[3]	Neutrophils <0.5 G/L[3]
yes, n (%)	39 (64)	50 (82)	44 (72)
no, n (%)	21 (34)	10 (16)	16 (26)
unknown (%)	1 (2)	1 (2)	1 (2)
Second cycle, n = 61	Platelets <100 G/L[4]	Neutrophils <1.0 G/L[4]	Neutrophils <0.5 G/L[4]
yes, n (%)	25 (41)	28 (46)	15 (25)
no, n (%)	34 (56)	31 (51)	44 (72)
unknown (%)	2 (3)	2 (3)	2 (3)

[3]Measured during the second cycle of venetoclax

[4]Measured at the time after the second cycle of venetoclax (at the start of the next cycle)

Supplemental Table S6: Association of venetoclax levels with hematologic regeneration and rate of CR.

		Total	CR[1]	no CR[1]	p-Value
Trough level, µg/L, n = 59	median				
	<1220, n (%)	29 (49)	16 (55)	13 (45)	0.61
	≥1220, n (%)	30 (51)	14 (47)	16 (53)	
	percentile 25%				
	<663, n (%)	15 (25)	7 (47)	8 (53)	0.77
	≥663, n (%)	44 (75)	23 (52)	21 (48)	
Peak level, µg/L (n = 43)	median				
	<2117, n (%)	21 (49)	8 (38)	13 (62)	0.76
	≥2117, n (%)	22 (51)	10 (45)	12 (55)	
	percentile 25%				
	<1177, n (%)	11 (26)	5 (45)	6 (55)	>0.99
	≥1177, n (%)	32 (74)	13 (41)	19 (59)	
		Total	Platelets >100 G/L[2]	Platelets <100 G/L[2]	p-Value
Trough level, µg/L (n = 59)	median				
	<1220, n (%)	29 (49)	15 (52)	14 (48)	0.80
	≥1220, n (%)	30 (51)	14 (47)	16 (53)	
	percentile 25%				
	<663, n (%)	15 (25)	6 (40)	9 (60)	0.55
	≥663, n (%)	44 (75)	23 (52)	21 (48)	
Peak level, µg/L (n = 43)	median				
	<2117, n (%)	21 (49)	10 (48)	11 (52)	0.76
	≥2117, n (%)	22 (51)	9 (41)	13 (59)	
	percentile 25%				
	<1177, n (%)	11 (26)	6 (55)	5 (45)	0.49
	≥1177, n (%)	32 (74)	13 (41)	19 (59)	
		Total	Neutrophils >1.0 G/L[2]	Neutrophils <1.0 G/L[2]	p-Value
Trough level, µg/L (n = 59)	median				
	<1220, n (%)	29 (49)	17 (59)	12 (41)	0.20
	≥1220, n (%)	30 (51)	12 (40)	18 (60)	
	percentile 25%				
	<663, n (%)	15 (25)	7 (47)	8 (53)	>0.99
	≥663, n (%)	44 (75)	22 (50)	22 (50)	
Peak level, µg/L (n = 43)	median				
	<2117, n (%)	21 (49)	10 (48)	11 (52)	0.54
	≥2117, n (%)	22 (51)	8 (36)	14 (64)	
	percentile 25%				
	<1177, n (%)	11 (26)	5 (45)	6 (55)	>0.99
	≥1177, n (%)	32 (74)	13 (41)	19 (59)	
		Total	Neutrophils >0.5 G/L[2]	Neutrophils <0.5 G/L[2]	p-Value
Trough level, µg/L (n = 59)	median				
	<1220, n (%)	29 (49)	19 (66)	10 (34)	0.43
	≥1220, n (%)	30 (51)	16 (53)	14 (47)	
	percentile 25%				
	<663, n (%)	15 (25)	9 (60)	6 (40)	>0.99
	≥663, n (%)	44 (75)	26 (59)	18 (41)	
Peak level, µg/L (n = 43)	median				
	<2117, n (%)	21 (49)	12 (57)	9 (43)	0.55
	≥2117, n (%)	22 (51)	10 (45)	12 (55)	
	percentile 25%				

	<1177, n (%)	11 (26)	5 (45)	6 (55)	0.74
	≥1177, n (%)	32 (74)	17 (53)	15 (47)	

[1]Complete remission

[2]Measured after first cycle of venetoclax (at the time of the start of the next cycle)

Supplemental Table S7: Association of venetoclax levels with sex.

		Male (n = 42)	Female (n = 33)	p-Value
Complete remission	yes, n (%)	29 (69)	20 (61)	
	no, n (%)	12 (29)	12 (36)	0.62
	unknown, n (%)	1 (2)	1 (3)	
Trough level, µg/L	median	1193	1151	
	min.	61	140	0.44
	max.	3722	3556	
Peak level, µg/L	median	2210	2025	
	min.	419	485	0.47
	max.	7849	4905	

Supplemental Table S8: Association of venetoclax levels with dose of venetoclax.

		400mg venetoclax (n = 17)	100mg venetoclax + azole antifungals (n = 58)	p-Value
Complete remission	yes, n (%)	14 (82)	35 (60)	
	no, n (%)	3 (18)	21 (36)	0.15
	unknown, n (%)	0 (0)	2 (3)	
Trough level, µg/L	median	1057	1219	
	min.	41	61	0.70
	max.	3799	5366	
Peak level, µg/L	median	2206	2091	
	min.	62	439	0.15
	max.	7849	4905	

Supplemental Table S9: Association of venetoclax levels with patient age at first diagnosis.

		Patients <70 years (n = 37)	Patients ≥70 years (n = 38)	p-Value
Complete remission	yes, n (%)	26 (70)	23 (61)	
	no, n (%)	11 (30)	13 (34)	0.62
	unknown, n (%)	0 (0)	2 (5)	
Trough level, µg/L	median	1163	1268	
	min.	140	61	0.2
	max.	3563	3722	
Peak level, µg/L	median	2056	2166	
	min.	4049	7849	0.06
	max.	419	800	

Supplemental Table S10: Association of venetoclax levels with the combination agent.

		Azacitidine, n = 65	Decitabine or cytarabine with/without cladribine, n = 10	p-Value
Complete remission	yes, n (%)	42 (65)	7 (70)	
	no, n (%)	21(32)	3 (30)	>0.99
	unknown, n (%)	2 (3)	0 (0)	
Trough level, µg/L	median	1252	870	
	min.	61	140	0.03
	max.	3722	2324	
Peak level, µg/L	median	2214	658	
	min.	547	419	0.001
	max.	7849	1997	
